# Supplementary figures and images for: Impact of lifestyle behaviors on the development of lifestyle diseases: A retrospective cohort study
Source: PLoS One. 2025 Jul 29;20(7):e0327505. doi: 10.1371/journal.pone.0327505 (PMC12306743; doi:10.1371/journal.pone.0327505)

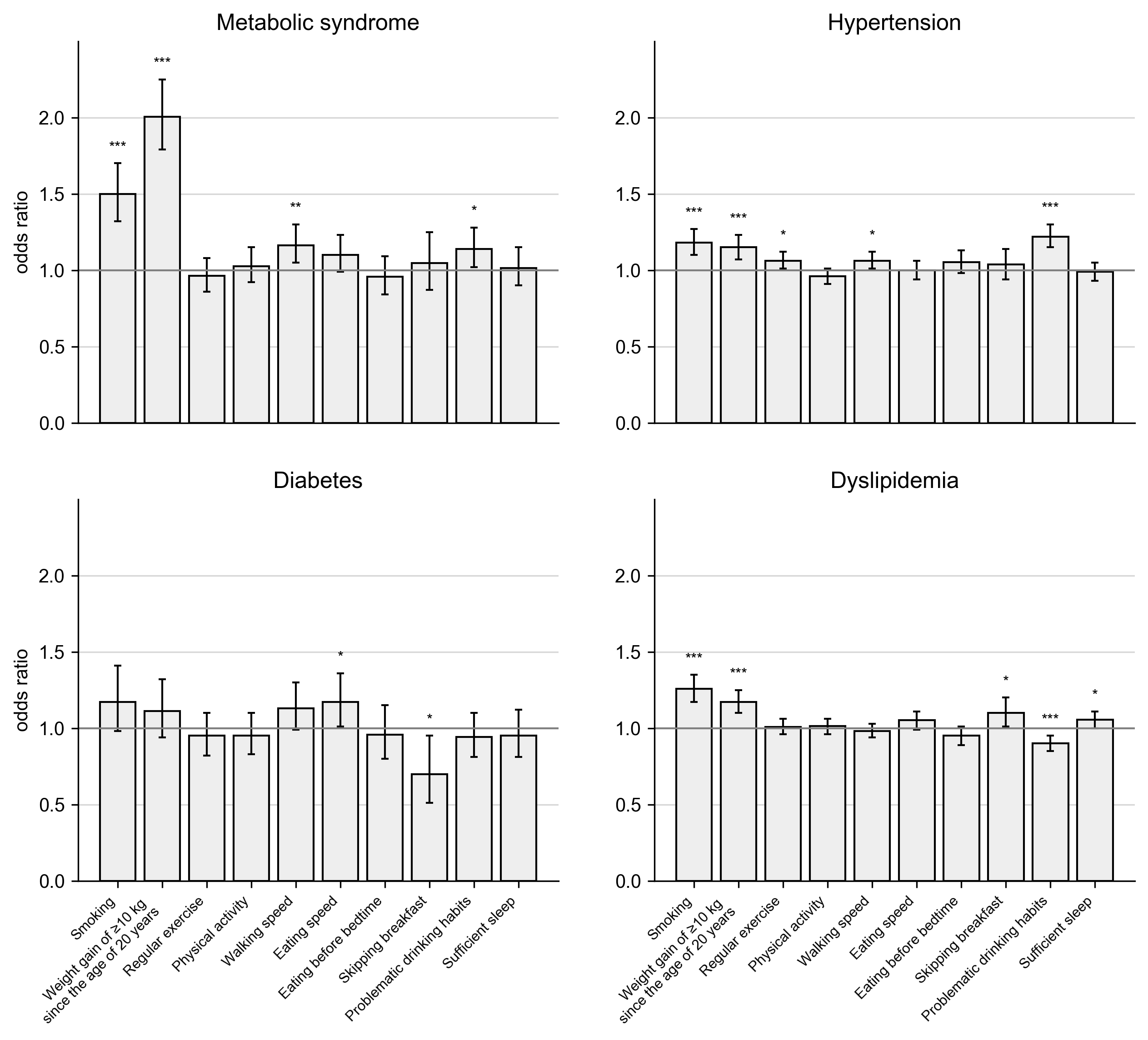

Supplement: S1 Fig — The odds ratio (black bars) with corresponding 95% confidence intervals (error bars) is shown for each category of lifestyle diseases. *P < 0.05, **P < 0.01, ***P < 0.001 compared with the reference category. (TIFF) [file pone.0327505.s004.tiff]
