# Supplementary material for: Impact of lifestyle behaviors on the development of lifestyle diseases: A retrospective cohort study
Source: PLoS One. 2025 Jul 29;20(7):e0327505. doi: 10.1371/journal.pone.0327505 (PMC12306743; doi:10.1371/journal.pone.0327505)
Supplement: Attachment — Submitted filename: COMMENTS ON ARTICLE.pdf [file pone.0327505.s005.pdf]

COMMENTS ON ARTICLE

IMPACT OF LIFESTYLE BEHAVIORS ON THE DEVELOPMENT OF LIFESTYLE DISEASES: A RETROSPECTIVE COHORT STUDY (PONE-D-24-56824)

NO	COMPONENT	COMMENT	SUGGESTION
1	Large dataset, which is very good	The large sample size enhances the study's statistical power and reliability.	Emphasize in the discussion how this study's sample size strengthens the validity of the findings.
2	Comprehensive analysis	The use of multivariate logistic regression models provides robust statistical insights into the associations between lifestyle behaviors and diseases.	Highlight how the inclusion of multiple adjusted models (Model 1 and Model 2) accounts for confounding variables.
3	Timeliness and relevance	The study aligns with global efforts to address the rising burden of NCDs.	Strengthen the introduction by framing the study's importance within the context of global public health priorities.
4	Generalizability	The study population is limited to Gunma Prefecture, Japan, potentially reducing the generalizability of the findings.	Discuss how similar studies in different populations or regions could validate the findings and make them applicable on a broader scale.
5	Self-reported data	The reliance on self-administered questionnaires introduces potential biases, such as recall or social desirability bias.	Mention strategies for mitigating these biases, such as corroborating self-reported behaviors with objective measures (e.g., wearable devices for physical activity).
6	Lack of longitudinal behavior tracking	The study does not account for changes in lifestyle behaviors over the follow-up period, which may influence the results.	Acknowledge this limitation explicitly and propose future research to incorporate longitudinal tracking of lifestyle changes.
7	Novel insights	The finding that skipping breakfast may protect against diabetes is intriguing but contradicts much	Expand the discussion on possible mechanisms or local dietary patterns that may explain this result. Recommend

		of the existing literature.	further studies to confirm or refute this finding.
8	Broader context	The manuscript would benefit from comparing findings with similar studies in different countries or regions.	Incorporate references to global studies that have examined similar relationships, emphasizing similarities and differences.
9	Clinical implications	While the statistical significance of the findings is clear, their clinical relevance needs more discussion.	Address how the results can guide practical recommendations for public health interventions or individual counseling.
10	Methodological details	Some criteria for defining "risky behaviors" are not sufficiently detailed.	Provide additional clarification on how lifestyle risks were categorized (e.g., thresholds for alcohol consumption).

General comments: I would say that the manuscript addresses an important public health issue by exploring the relationship between lifestyle behaviors and lifestyle diseases using a large dataset. The findings have potential implications for public health policies and preventive interventions. However, several aspects of the study, including its generalizability, methodological limitations, and the depth of the discussion, need further elaboration.

Apart from that, the abstract could benefit from simplification by highlighting the most impactful findings and their broader implications, ensuring readers quickly grasp the study's significance.

Then, in the discussion, greater emphasis should be placed on the public health relevance of targeting smoking and weight gain, as these are common risk factors across multiple diseases and could serve as key areas for intervention. Additionally, the conclusion would be strengthened by addressing the study's limitations more explicitly, particularly the need for validation studies in diverse populations to enhance the generalizability of the findings.
