# Supplementary material for: Impact of lifestyle behaviors on the development of lifestyle diseases: A retrospective cohort study
Source: PLoS One. 2025 Jul 29;20(7):e0327505. doi: 10.1371/journal.pone.0327505 (PMC12306743; doi:10.1371/journal.pone.0327505)
Supplement: Attachment — Submitted filename: Response to Reviewers.docx [file pone.0327505.s007.docx]

Dear editors,

This is our response to the comments of the referees to PONE-D-24-56824,

Impact of Lifestyle Behaviors on the Development of Lifestyle Diseases: A Retrospective Cohort Study

by Takafumi Okawa *et al.*

Thank you for your continued consideration of our manuscript for publication in *PLOS ONE*. We sincerely appreciate the time and effort that you and the reviewers have dedicated to evaluating our work. Based on the valuable feedback received, we have carefully revised the manuscript, and we believe these revisions have strengthened its quality.

Below, we provide detailed responses to the reviewers’ and your comments.

We look forward to your editorial decision.

Here is a list of changes.

**EDITOR’S SPECIFIC COMMENTS:**

1. **Large dataset, which is very good**

**The large sample size enhances the study's statistical power and reliability. Emphasize in the discussion how this study’s sample size strengthens the validity of the findings.**

-Response-

We appreciate the editor’s positive feedback. As suggested, we have enhanced the Discussed section by clarifying this information (lines 207-208) as follows:

“To our knowledge, this study is the first to assess the impact of lifestyle behaviors on the development of lifestyle diseases using such an extensive dataset, which enhances the statistical power and reliability of the findings.”

2. **Comprehensive analysis**

**The use of multivariate logistic regression models provides robust statistical insights into the associations between lifestyle behaviors and diseases. Highlight how the inclusion of multiple adjusted models (Model 1 and Model 2) accounts for confounding variables.**

-Response-

We thank the editor for the positive comment. To improve clarity, we have added the Discussed section by clarifying this information (lines 211-212) as follows:

“New insights were obtained using a two-stage multivariable logistic regression model, where Model 1 and Model 2 were sequentially adjusted to account for potential confounding variables.”

3. **Timeliness and relevance**

**The study aligns with global efforts to address the rising burden of NCDs. Strengthen the introduction by framing the study's importance within the context of global public health priorities.**

-Response-

We appreciate the editor’s positive feedback. As suggested, we have enhanced the Introduction section by clarifying this information and included the relevant article in the References (lines 48-50) as follows:

“The increase in non-communicable diseases (NCDs) with the aging population has led to a rise in national healthcare expenditures, making cost containment a critical public health priority [1-3].”

We have added the following references:

1.Curtis LH, Hammill BG, Bethel MA, Anstrom KJ, Gottdiener JS, Schulman KA. Costs of the metabolic syndrome in elderly individuals: findings from the Cardiovascular Health Study. Diabetes Care. 2007;30(10):2553-8. Epub 20070710. doi: 10.2337/dc07-0460. PubMed PMID: 17623825.

2.Muka T, Imo D, Jaspers L, Colpani V, Chaker L, van der Lee SJ, et al. The global impact of non-communicable diseases on healthcare spending and national income: a systematic review. Eur J Epidemiol. 2015;30(4):251-77. Epub 20150118. doi: 10.1007/s10654-014-9984-2. PubMed PMID: 25595318.

3.Nishida Y, Anzai T, Takahashi K, Kozuma T, Kanda E, Yamauchi K, et al. Multimorbidity patterns in the working age population with the top 10% medical cost from exhaustive insurance claims data of Japan Health Insurance Association. PLoS One. 2023;18(9):e0291554. Epub 20230928. doi: 10.1371/journal.pone.0291554. PubMed PMID: 37768909; PubMed Central PMCID: PMCPMC10538783.

4. **Generalizability**

**The study population is limited to Gunma Prefecture, Japan, potentially reducing the generalizability of the findings. Discuss how similar studies in different populations or regions could validate the findings and make them applicable on a broader scale.**

-Response-

As requested, we have revised this information to the Discussion section (lines 285-287) in response to reviewer A’s comment #4 and reviewer B’s comment #5.

5. **Self-reported data**

**The reliance on self-administered questionnaires introduces potential biases, such as recall or social desirability bias. Mention strategies for mitigating these biases, such as corroborating self-reported behaviors with objective measures (e.g., wearable devices for physical activity).**

-Response-

As requested, we have revised this information to the Discussion section (lines 278-283) in response to reviewer A’s comment #3 and reviewer B’s comment #5.

6. **Lack of longitudinal behavior tracking**

**The study does not account for changes in lifestyle behaviors over the follow-up period, which may influence the results. Acknowledge this limitation explicitly and propose future research to incorporate longitudinal tracking of lifestyle changes.**

-Response-

As requested, we have revised this information to the Discussion section (lines 276-278) in response to reviewer A’s comment #3.

7. **Novel insights**

**The finding that skipping breakfast may protect against diabetes is intriguing but contradicts much of the existing literature. Expand the discussion on possible mechanisms or local dietary patterns that may explain this result. Recommend further studies to confirm or refute this finding.**

-Response-

As requested, we have revised this information to the Discussion section (lines 228-233) in response to reviewer A’s comment #2 and reviewer B’s comment #7.

8. **Broader context**

**The manuscript would benefit from comparing findings with similar studies in different countries or regions. Incorporate references to global studies that have examined similar relationships, emphasizing similarities and differences.**

-Response-

We appreciate the reviewer’s suggestion to compare our findings with similar studies conducted in different countries or regions. As suggested, we have incorporated references to global studies that have examined similar associations and have discussed both similarities and differences. The revised text has been added to the Discussion section (lines 269-273) as follows:

“The impact of lifestyle factors such as smoking, weight management, physical activity, and alcohol consumption on cardiovascular disease and life expectancy has been widely recognized across different populations [45, 46]. However, cultural and dietary differences may influence these associations. Our study, which adjusted for 10 detailed lifestyle habits, offers a broader perspective than previous studies. Further research in diverse populations is needed to confirm these findings.”

We have added the following references:

45.Li Y, Schoufour J, Wang DD, Dhana K, Pan A, Liu X, et al. Healthy lifestyle and life expectancy free of cancer, cardiovascular disease, and type 2 diabetes: prospective cohort study. BMJ. 2020;368:l6669. Epub 20200108. doi: 10.1136/bmj.l6669. PubMed PMID: 31915124; PubMed Central PMCID: PMCPMC7190036.

46.Su J, Geng H, Chen L, Fan X, Zhou J, Wu M, et al. Association of healthy lifestyle with incident cardiovascular diseases among hypertensive and normotensive Chinese adults. Front Cardiovasc Med. 2023;10:1046943. Epub 20230302. doi: 10.3389/fcvm.2023.1046943. PubMed PMID: 36937945; PubMed Central PMCID: PMCPMC10017485.

9. **Clinical implications**

**While the statistical significance of the findings is clear, their clinical relevance needs more discussion. Address how the results can guide practical recommendations for public health interventions or individual counseling.**

-Response-

As requested, we have revised this information to the Discussion section (lines 264-268) in response to reviewer A’s comment #1 and reviewer B’s comment #6.

10. **Methodological details**

**Some criteria for defining "risky behaviors" are not sufficiently detailed. Provide additional clarification on how lifestyle risks were categorized (e.g., thresholds for alcohol consumption).**

-Response-

We appreciate the opportunity to clarify this point. The presence or absence of lifestyle behavior risks is already detailed in S2 Table. Additionally, the criteria for determining drinking risk, based on the frequency of drinking (Question 9) and the amount of alcohol consumed (Question 10), are described in the Methods section (lines 120-127), following previous studies [19]. We believe these details sufficiently address the editor’s concern.

**EDITOR’S General comments:**

-Response-

We appreciate the editor’s suggestion regarding the abstract. To improve clarity and ensure that readers quickly grasp the study's significance, we have simplified the abstract by emphasizing the most impactful findings and their broader implications, in response to Reviewer B’s comment #4.

Although not directly related to the reviewer’s comments, we have updated the Acknowledgments section to accurately reflect contributions to this study.

Response to Reviewer A

1. **The statistical analysis is rigorous, employing multivariate models that explore the independent effects of lifestyle behaviors. However, additional details on multicollinearity diagnostics, such as reporting variance inflation factors (VIFs), would further validate the results. While the use of odds ratios with confidence intervals is appropriate, the discussion could benefit from emphasizing the clinical relevance of statistically significant findings. For instance, while smoking and weight gain emerged as common risk factors across relevance of statistically significant findings. For instance, while smoking and weight gain emerged as common risk factors across multiple diseases, it would be helpful to contextualize these findings within public health interventions.**

-Response-

We appreciate the reviewer’s insightful comments on the statistical analysis and the clinical relevance of our findings.

To address concerns regarding multicollinearity, we have calculated the variance inflation factors (VIFs) for all independent variables and confirmed that all values are below 5, indicating that multicollinearity is not a concern in our models. This information has been added to the Methods section (lines 148-150) as follows:

“Correlation coefficients and variance inflation factors (VIFs) were calculated for each variable to assess multicollinearity among the independent variables, confirming that all VIF values were below 5.”

Additionally, we have revised the Discussion section to emphasize the clinical relevance of our findings. Specifically, we have highlighted the importance of smoking and weight gain as common risk factors across multiple diseases and discussed their implications for public health interventions. The revised text has been added to the Discussion section (lines 264-268) as follows:

“The findings of this study provide essential evidence to support and strengthen targeted public health interventions, such as smoking cessation programs and weight management strategies based on the Health Promotion Act. Addressing these two factors through such interventions could play a crucial role in disease prevention and risk reduction.”

We believe these revisions strengthen the manuscript by providing a clearer interpretation of the findings and their public health significance.

2. **One intriguing finding is the protective association between skipping breakfast and diabetes, which contradicts much of the existing literature. While this result is novel, it warrants further exploration, possibly through sensitivity or subgroup analyses to account for regional dietary habits or meal composition. The manuscript would also benefit from a discussion on residual confounding, as factors like socioeconomic status or genetic predispositions were not included in the analysis.**

-Response-

We thank the reviewer for highlighting this important issue regarding the association between skipping breakfast and diabetes. As noted, our findings indicate a protective association, which is indeed contrary to the majority of existing studies in the field. We recognize that this result requires further investigation. To address this point, we have expanded our discussion in more detail. This information has been added to the Discussion section (lines 228-233) as follows:

“According to the NHNS in 2016, despite similar daily energy intake, the residents of Gunma Prefecture did consume significantly more carbohydrates compared with the national average, potentially affecting glucose metabolism and insulin sensitivity. Additionally, meal timing and overall dietary patterns could play a role. A previous study suggest that prolonged fasting intervals may improve metabolic health by enhancing insulin sensitivity and glycemic control　[32]. It is possible that individuals who skip breakfast in this population may compensate with well-balanced meals later in the day, mitigating the adverse effects typically associated with breakfast skipping. Therefore, further studies are needed to examine the relationship between skipping breakfast and diabetes onset, taking into account both calorie intake and meal composition.”

We have added the following references:

32.Sutton EF, Beyl R, Early KS, Cefalu WT, Ravussin E, Peterson CM. Early Time-Restricted Feeding Improves Insulin Sensitivity, Blood Pressure, and Oxidative Stress Even without Weight Loss in Men with Prediabetes. Cell Metab. 2018;27(6):1212-21 e3. Epub 20180510. doi: 10.1016/j.cmet.2018.04.010. PubMed PMID: 29754952; PubMed Central PMCID: PMCPMC5990470.

3. **Although the reliance on self-reported data is acknowledged as a limitation, it introduces potential biases such as recall and social desirability bias. A more detailed discussion on how these biases may have influenced the results would strengthen the manuscript. Additionally, the study does not account for changes in lifestyle behaviors during the follow-up period, which may affect the observed associations. Future research should consider longitudinal tracking of behavioral changes to provide a more nuanced understanding of their effects.**

-Response-

We appreciate the reviewer’s precise comments regarding potential biases introduced by self-reported data and the lack of lifestyle behavior tracking during the follow-up period. As suggested, we have expanded the Discussion section (lines 278-283) to provide a more detailed discussion on the potential influence of recall and social desirability bias, as follows:

“Second, the lifestyle assessment data collected using self-administered questionnaires lack objectivity and are subject to potential biases, such as recall bias and social desirability bias. However, this method has been validated in studies by Takeuchi *et al*.[47] and Fukasawa *et al*.[19], demonstrating its statistical validity. To mitigate potential biases, future studies should consider complementing self-reported data with objective measures, such as wearable devices for physical activity monitoring.”

Additionally, we have addressed the limitation of not accounting for lifestyle changes over the follow-up period. The revised text is included in the Discussion section (lines 276-278) as follows:

“Longitudinal studies often struggle to incorporate the changes in background factors into their models; therefore, future research incorporating longitudinal tracking of lifestyle behaviors to account for these changes is necessary.”

4. **In terms of generalizability, the study is limited to a specific region in Japan, and the findings may not be directly applicable to other populations with different cultural, dietary, or healthcare contexts. The authors should discuss how similar studies in other regions could validate and expand upon these results.**

-Response-

We appreciate the reviewer’s comment regarding the generalizability of our findings. As suggested, we have expanded the Discussion section (lines 285-287) to acknowledge the study’s regional limitations and emphasize the need for validation in diverse populations. The added text is as follows:

“Regional characteristics, economic indicators, and the social determinants of health may influence the outcomes. Future studies in diverse populations and healthcare systems are needed to validate these findings and ensure broader applicability.”

Response to Reviewer B

1. **Explain why the analysis excluded individuals who had multiple checkups. If this is done, selection bias could be introduced into the analysis because such persons may differ in some systematic way from those included in the analysis.**

-Response-

We appreciate the opportunity to clarify this point. The analysis excluded individuals who had multiple checkups to maintain the independence of observations and avoid potential bias introduced by repeated measurements. While we recognize that excluding these individuals could introduce selection bias, we believe that their inclusion could have led to an overrepresentation of certain patterns in the data, as individuals with multiple checkups may have characteristics that differ systematically from those who had only one checkup. We also acknowledge that future studies could explore the effect of including individuals with multiple checkups in order to better understand any associated biases.

2. **Address the multicollinearity issue in the logistic regression models by reporting the VIF values and discussing how the overlapping lifestyle behaviors were dealt with.**

-Response-

We appreciate the reviewer’s comment regarding multicollinearity in the logistic regression models. We recognize that this is an important concern, as it has been raised by multiple reviewers. To address concerns regarding multicollinearity, we have calculated the variance inflation factors (VIFs) for all independent variables and confirmed that all values are below 5, indicating that multicollinearity is not a concern in our models. This information has been added to the Methods section (lines 148-150) as follows:

“Correlation coefficients and variance inflation factors (VIFs) were calculated for each variable to assess multicollinearity among the independent variables, confirming that all VIF values were below 5.”

3. **Simplify and visualize results using plots of bar charts or heat maps that give in one glance several key findings of Table 1 and Table 2.**

-Response-

We appreciate the reviewer’s suggestion to simplify and visualize key findings from the tables. In response, we have created a new bar chart to provide a clearer overview of the results. This figure has been added as Figure S1 and we have revised the Result section (lines185-186) as follows:

“The results of Model 2, adjusted for sex, age, BMI, and laboratory values, are presented below (see Supplementary Figure S1).”

We have added this to the Supporting Information section (lines 300-303) as follows:

“S1 Figure. Correlation between lifestyle behaviors and lifestyle diseases determined using a multivariable logistic regression model (Supplementary to Table 2). The odds ratio (black bars) with corresponding 95% confidence intervals (error bars) is shown for each category of lifestyle diseases. *P<0.05, **P<0.01, ***P<0.001 compared with the reference category. (TIFF)”

4. **The findings should be discussed for their clinical significance. However, not every statistically significant association is clinically significant and may deserve further consideration in terms of modest effect size, such as the identified one for fast eating and diabetes, with an OR of 1.17.**

-Response-

We appreciate the reviewer’s important observation regarding the clinical significance of our findings. This important point was not clear in the former text. To address this concern, we have revised the abstract (lines 38-40) to explicitly highlight the strongest association observed in our study and that smoking and weight gain were identified as common risk factors for MetS, hypertension, and dyslipidemia. The revised text is as follows:

“In this study, weight gain of ≥10 kg since the age of 20 years showed the strongest association with MetS (OR: 2.01; 95% CI, 1.79–2.25). Additionally, smoking and weight gain were identified as common risk factors for MetS, hypertension, and dyslipidemia.”

5. **Expand the discussion of limitations. Acknowledge potential biases (e.g., recall bias, selection bias) and the limited generalizability of the findings.**

-Response-

We appreciate the reviewer’s comment regarding the need to expand the discussion of limitations. We recognize that this is an important concern, as it aligns with Reviewer A’s comments #3 and #4. As suggested, we have revised the Discussion section (lines 278-287) to explicitly acknowledge potential biases, including recall bias and selection bias, as well as the limited generalizability of our findings. The revised text is as follows:

“Second, the lifestyle assessment data collected using self-administered questionnaires lack objectivity and are subject to potential biases, such as recall bias and social desirability bias. However, this method has been validated in studies by Takeuchi *et al*.[44] and Fukasawa *et al*.[19], demonstrating its statistical validity. To mitigate potential biases, future studies should consider complementing self-reported data with objective measures, such as wearable devices for physical activity monitoring. Third, the database used comprised information of individuals insured under the Gunma Prefecture’s NHI. Consequently, it remains unclear whether these findings can be generalized to other regions or insurance groups. Regional characteristics, economic indicators, and the social determinants of health may influence the outcomes. Future studies in diverse populations and healthcare systems are needed to validate these findings and ensure broader applicability.”

6. **Highlight practical implications: Describe how such findings might be used to inform public health interventions or policy.**

-Response-

We appreciate the reviewer’s suggestion to highlight the practical implications of our findings, as this point has also been raised by other reviewers. We have revised the Discussion section to emphasize the clinical relevance of our findings. Specifically, we have highlighted the importance of smoking and weight gain as common risk factors across multiple diseases and discussed their implications for public health interventions. The revised text has been added to the Discussion section (lines 264-268) as follows:

“The findings of this study provide essential evidence to support and strengthen targeted public health interventions, such as smoking cessation programs and weight management strategies based on the Health Promotion Act. Addressing these two factors through such interventions could play a crucial role in disease prevention and risk reduction.”

We believe these revisions strengthen the manuscript by providing a clearer interpretation of the findings and their public health significance.

7. **The negative relation of breakfast omission with diabetes appears intriguingly protective, with an odds ratio of 0.70, which also goes against previous reports. This might be discussed more elaborately in the discussion part, considering either regional dietary habits or confounding.**

-Response-

We thank the reviewer for the thoughtful comment regarding the negative association between breakfast omission and diabetes. As this is an important point that aligns with Reviewer A’s comment #2, our findings indicate a protective association, which is indeed contrary to the majority of existing studies in the field. We recognize that this result requires further investigation. To address this point, we have expanded our discussion to explore potential factors, such as regional dietary habits and possible confounding variables, that may help explain this unexpected finding. This information has been added to the Discussion section (lines 228-233) as follows:

“According to the NHNS in 2016, despite similar daily energy intake, the residents of Gunma Prefecture did consume significantly more carbohydrates compared with the national average, potentially affecting glucose metabolism and insulin sensitivity. Additionally, meal timing and overall dietary patterns could play a role. A previous study suggest that prolonged fasting intervals may improve metabolic health by enhancing insulin sensitivity and glycemic control　[32]. It is possible that individuals who skip breakfast in this population may compensate with well-balanced meals later in the day, mitigating the adverse effects typically associated with breakfast skipping. Therefore, further studies are needed to examine the relationship between skipping breakfast and diabetes onset, taking into account both calorie intake and meal composition.”

We have added the following references:

32.Sutton EF, Beyl R, Early KS, Cefalu WT, Ravussin E, Peterson CM. Early Time-Restricted Feeding Improves Insulin Sensitivity, Blood Pressure, and Oxidative Stress Even without Weight Loss in Men with Prediabetes. Cell Metab. 2018;27(6):1212-21 e3. Epub 20180510. doi: 10.1016/j.cmet.2018.04.010. PubMed PMID: 29754952; PubMed Central PMCID: PMCPMC5990470.

8. **Where relevant, for example, when referring to the "standard questionnaire" or cutoff values, the manuscript should refer explicitly to supplementary tables.**

-Response-

We thank the reviewer for the careful review of the manuscript. Our previous expression was unclear and may have led to a misinterpretation. We have revised the Materials and Methods section (lines 115-120) to ensure that the reference to Supplementary Table S2 is accurate and that the supplementary tables are explicitly cited when mentioning the “standard questionnaire” and cutoff values, as follows:

“Age, BMI, and laboratory values were categorized according to the medical guidelines, as detailed in S1 Table. Data on lifestyle behaviors at the initial checkup were extracted from the results of the “standard questionnaire” provided by the MHLW. From this questionnaire, 11 questions that have consistently provided valid responses over the years were selected (as shown in S2 Table). The presence or absence of lifestyle behavior risks is also presented in S2 Table.”
