# Supplementary material for: Impact of lifestyle behaviors on the development of lifestyle diseases: A retrospective cohort study
Source: PLoS One. 2025 Jul 29;20(7):e0327505. doi: 10.1371/journal.pone.0327505 (PMC12306743; doi:10.1371/journal.pone.0327505)
Supplement: Attachment — Submitted filename: Response to Revie wer_ver2.docx [file pone.0327505.s008.docx]

Dear editors,

This is our response to the comments of the referees to PONE-D-24-56824,

Impact of Lifestyle Behaviors on the Development of Lifestyle Diseases: A Retrospective Cohort Study

by Takafumi Okawa *et al.*

Thank you for your continued consideration of our manuscript for publication in *PLOS ONE*. We sincerely appreciate the time and effort that you and the reviewers have dedicated to evaluating our work. Based on the valuable feedback received, we have carefully revised the manuscript, and we believe these revisions have strengthened its quality.

Below, we provide detailed responses to the reviewers’ and your comments.

We look forward to your editorial decision.

Here is a list of changes.

Response to Reviewer

1. **However, I want to voice my concern over the findings' interpretability and generalizability. The implication that skipping breakfast is linked to less diabetes contradicts the literature. It suggests a more elaborate sensitivity analysis or other explanations, such as reverse causation or bias for reporting. Although the data are extensive, the sample is geographically and demographically restricted (only insured adults within Gunma Prefecture), and this may be better highlighted. The confounding effect from unmeasured lifestyles or socioeconomic factors (e.g., sleep, work, job status) could also be mentioned.**

-Response-

We appreciate your valuable comments and thoughtful suggestions. We fully understand your concerns regarding the interpretability and generalizability of our findings. As suggested, we have carefully revised the Discussion section to explicitly acknowledge and elaborate on the following points:

1) The possibility of reverse causation (e.g., individuals with early-stage diabetes may have skipped breakfast to manage symptoms).
2) The potential impact of reporting bias due to self-reported dietary habits.
3) The limited generalizability of our findings, as the study population included only insured adults in Gunma Prefecture.
4) The likelihood of residual confounding from unmeasured lifestyle or socioeconomic factors (e.g., sleep patterns, job status).

These points have now been fully integrated into the Discussion section (lines 233–241) to provide a balanced interpretation of the findings and to caution against overgeneralization.

“Nonetheless, alternative explanations should be considered. For example, reverse causation may partly explain this finding—for instance, individuals with preclinical diabetes may have modified their eating habits by skipping breakfast to manage symptoms or weight. In addition, since dietary habits were self-reported, reporting bias may have influenced the observed association. Furthermore, our study was limited to insured adults within Gunma Prefecture, which may restrict the generalizability of our findings. Finally, unmeasured confounders such as sleep patterns, work schedules, and socioeconomic status could also have affected the results. Taken together, these factors highlight the need for cautious interpretation of our findings and suggest that further studies using objective dietary assessments and longitudinal data are warranted to clarify these associations.”

2. **Finally, whilst the manuscript is composed well and readable, the Data Availability Statement is inconsistent with PLOS’s open data policy. Please specify a means for de-identified data availability through a recognized repository or institutional arrangements. I suggest minor revisions and changes to meet these issues and enhance the manuscript's contribution to the evidence base for preventive health actions through behavioral data.**

-Response-

We appreciate your important comment regarding data availability. As discussed with the editorial office, the health check-up data used in this study contain sensitive personal health information and are subject to ethical and legal restrictions that prevent full public sharing.

However, in accordance with PLOS ONE’s data policy, we have clarified in the revised Data Availability Statement that de-identified data may be made available upon reasonable request and subject to approval by the Ethics Committee for Research Involving Humans at the Gunma University Faculty of Medicine.

This committee is an independent body that oversees ethical compliance for human research at Gunma University. Importantly, none of the authors are affiliated with the committee or have access to its administrative functions, ensuring impartiality in the data access process.

To support transparency and facilitate data access within ethical constraints, we have provided a non-author contact email for the committee's administrative office (hitotaisho-ciru@ml.gunma-u.ac.jp). The updated statement has been added to the revised manuscript in the Data Availability section (lines 310–313) as follows:

"Requests for data access should be directed to the Office of the Ethics Committee for Research Involving Humans, Advanced Medical Development Center, Gunma University (Email: [hitotaisho-ciru@ml.gunma-u.ac.jp](mailto:hitotaisho-ciru@ml.gunma-u.ac.jp)). These data are available for researchers who meet the criteria for access to confidential data as determined by the ethics committee."

This arrangement ensures both compliance with ethical and legal requirements and the availability of data under controlled access. The revised statement has been reviewed and approved by the PLOS editorial office to ensure alignment with the journal’s open data policy. We hope this revision addresses the reviewer’s concern and affirms our commitment to transparency and research integrity.
